# Synthesis and Characterization of Cellulose Microfibril-Reinforced Polyvinyl Alcohol Biodegradable Composites

**DOI:** 10.3390/ma17020526

**Published:** 2024-01-22

**Authors:** Fatemeh Mahdiyeh Boroujeni, Gabriella Fioravanti, Ronald Kander

**Affiliations:** School of Design and Engineering, Kanbar College, Thomas Jefferson University, Philadelphia, PA 19144, USA; fatemeh.mahdiyeh-boroujeni@students.jefferson.edu (F.M.B.); gabriella.fioravanti@jefferson.edu (G.F.)

**Keywords:** biomass, cellulose nanocrystals, cellulose microfibrils, organic acid, biocomposites, polyvinyl alcohol, biodegradable, sustainability

## Abstract

The pursuit of an environmentally sustainable manufacturing process requires the substitution of less damaging and recyclable solutions for harmful reagents. This study aims to assess the effectiveness of using cellulose microfibrils synthesized via different hydrolysis reactions as reinforcing agents in polyvinyl alcohol (PVA) at varying concentrations. The investigation explores the morphology, thermal properties, and chemical behavior of the cellulose particles. The cellulose microfibrils (CMFs) produced using citric acid exhibited the highest yield and aspect ratio. Notably, particles from organic acids demonstrated greater thermal stability, with oxalic acid-derived particles displaying the maximum thermal degradation temperature. Subsequently, cast films of PVA reinforced with the cellulose microfibrils underwent comprehensive analyses, including Fourier transfer infrared (FTIR) spectroscopy, thermal degradation temperature (T_d_), differential scanning calorimetry (DSC), and tensile strength tests. The thermal behavior of cast films experienced notable changes with the addition of cellulose particles, evidenced by increased melting and crystallinity temperatures, along with a rise in the degree of crystallinity. The incorporation of cellulose particles led to a substantial improvement in mechanical properties. Films containing CMF displayed higher Young’s modulus, and the sample incorporating 5% CMF derived from citric acid exhibited the most significant increase in modulus.

## 1. Introduction

The emergence and progression of natural fiber-reinforced polymer composites is viewed as a promising environmentally friendly alternative to petrochemical-based, non-degradable polymeric materials [1,2]. Concerns related to synthetic fillers include high cost, non-biodegradability, non-combustibility, and challenges associated with sustainable recycling techniques [3]. Due to these concerns, there is a focus on exploring alternative bio-based fillers. Substituting synthetic fillers with natural ones, such as cellulose fibers, offers numerous advantages. Cellulose whiskers, with their high aspect ratio, low density, cost-effectiveness, low toxicity, excellent thermal and mechanical properties, biocompatibility, and biodegradability, have gained significant attention [4]. These whiskers can be synthesized from various materials such as wood, natural fibers, fungi, and algae. Natural fibers such as cotton, flax, bamboo, jute, and hemp exhibit relatively high strength, stiffness, and low density, though their properties are influenced by factors such as geography, climate, and plant age [5].

Hemp, the industrial *Cannabis sativa* varietal, is gaining popularity as an industrial biomass due to its durability, cost-effectiveness, biodegradability, and sustainability. It is a fast-growing crop requiring less water and fewer pesticides than other crops [6,7,8]. Recent studies have explored the environmental impact and properties of hemp, revealing its superiority in terms of sustainability over other forms of cellulose biomass (such as wood, cotton, and corn stover) requiring less water, fertilizers, herbicides, and insecticides while being cultivatable in a wide range of climates and growing conditions [9,10]. Currently, U.S.-manufactured cellulose nanomaterials are solely supplied by three main facilities, GranBio, Forest Products Lab, and University of Maine, and are predominantly manufactured from wood pulp [11].

Cellulose nanocrystals (CNCs), derived from hemp, are produced through acid hydrolysis of non-crystalline cellulose in the bast fibers using concentrated mineral acids, typically sulfuric acid. However, this method has drawbacks, such as high environmental impact, recovery difficulties, low thermal stability, challenges in functionalization due to sulfate groups, toxicity, and low CNC yield [12,13]. To address environmental concerns, there is a growing interest in replacing mineral acids with organic acids for CNC preparation [14]. Organic acids are preferred for their lower acidity, reduced corrosivity, and recyclability [15,16]. Additionally, during hydrolysis, organic acids such as oxalic acid, maleic acid, and citric acid can introduce functional groups like ester and carboxylic groups, enhancing surface functionality and resulting in improved thermal and mechanical properties [17,18]. Citric acid, derived from citrus fruits, is particularly notable for its minimal impact on human health and the environment, making it a safer and more sustainable choice [17]. Oxalic acid, produced from biomass and recoverable at low temperatures through crystallization, also contributes to sustainable and economical manufacturing [19].

CNCs, with their large surface area and abundant surface hydroxyl groups, serve as excellent reinforcing nanofillers for polymer biocomposites, offering high tensile strength and Young’s modulus [20]. Achieving good dispersion in the polymer matrix is essential for enhanced performance compared to the unreinforced polymer. Cellulose whiskers, with their hydrophilicity and OH surface groups, can form hydrogen bonds with polar polymers, producing high-performance biocomposites. This property enables better dispersion and interaction with polymers having hydroxyl groups, leading to effective polar surface interactions. By performing hydrolysis with oxalic or citric acid, the resulting functionality of the experimental CNCs will be different, thus yielding different interfacial tension and dispersion within polymer matrices. For incorporation of standard CNCs into non-polar polymer matrices, surface modification of the cellulose particles can be beneficial [21]. PVA, a highly polar polymer, establishes strong hydrogen-bonding interactions with CNCs with hydroxyl groups. The effective preparation of PVA/CNC biocomposites using an aqueous mixing process has been reported in the literature [22,23,24,25].

PVA has emerged as an environmentally friendly and practical alternative to petroleum-based polymers like polyethylene and polypropylene, as highlighted by Arindam in 2018 [25]. Its increasing prominence in various global commercial applications, such as packaging, clothing, printing, and medicine, is attributed to its distinctive chemical and physical properties [26,27,28]. PVA is known for being non-toxic, semicrystalline, and water-soluble, as emphasized by Lu in 2008 and Roohani in 2008 [29,30]. While recognized for biodegradability, its main drawback is the relatively high cost, which can be mitigated by blending it with other natural polymers or fillers. The incorporation of cellulose nanocrystals (CNCs) into PVA to create composites enhances polymer biodegradability, mechanical properties, and crystallinity and is supported by researchers worldwide [22,23,24,25].

Considering principles of sustainability, this work aims to replace sulfuric acid with organic acids (citric acid and oxalic acid) during manufacturing to address environmental concerns. This work will compare CNC yielded from wood and CNC yielded from hemp, outlining the morphology as well as the chemical and thermal properties of the cellulose whiskers. Additionally, the study investigates the effect of incorporating these fillers into a PVA polymer matrix at 1% and 5% filler loadings.

## 2. Materials and Methods

### 2.1. Materials

Degummed hemp fiber was acquired from Hemp Trader, Los Angeles, CA, USA (Product Code: F-DG1), Sulfuric acid (98%) was purchased from Sigma-Aldrich (St. Louis, MO, USA), Citric acid (Electrophoresis Grade, 99.5%) was obtained from ThermoFisher (Waltham, MA, USA), Oxalic acid (anhydrous, 98%) was provided from ThermoFisher, PVA (87–89% hydrolyzed, M.W.13,000–23,000) was obtained from Arcos Organics (Waltham, MA, USA), and through Fisher Scientific, cellulose nano crystals extracted from wood with sulfuric acid were purchased from the University of Maine (Orono, ME, USA) (https://umaine.edu/pdc/nanocellulose/, accessed on 15 January 2024) and used as a control in this study.

### 2.2. Cellulose Whisker Synthesis

Cellulose nano and micro particles were synthesized using an acid hydrolysis process, following the method described by Revol et al. [31]. The best yield was determined based on the literature, considering factors such as acid concentration, process temperature, and duration, which varied depending on the type of acid used [17,20,32].

Five grams of oven-dried degummed hemp fibers, with diameters ranging from approximately 5 to 80 microns, were cut into lengths of 0.5 cm to 1 cm. These prepared fibers were placed into three-necked, round-bottomed flasks, along with 50 mL of acid and distilled water, under constant stirring. In the case of sulfuric acid hydrolysis, the process was conducted using a 64% concentration at 65–70 °C for 60 min. The reaction was terminated by adding 400 mL distilled water. Subsequently, the suspension was centrifuged three times with DI water in an Eppendorf Centrifuge 5810 R for 15 min at 9289× *g* gravitational constant (g). The process continued with dialysis to remove the acid from suspension, adjusting the pH to 6–7. For sulfuric acid hydrolysis, this dialysis step extended over a period of 6–7 days. After dialysis, the suspension was recentrifuged one time to separate the particles into two phases: a clear liquid containing CNCs (cellulose nano crystals) and a liquid with larger agglomerated CMFs (cellulose micro fibrils).

The hydrolysis process for citric acid followed a similar protocol, with a slight variation. It involved the use of 80% citric acid and was conducted at a temperature of 95 °C to 105 °C with a duration of at least four hours, until all fibers were completely dissolved. Following hydrolysis, 400 mL of hot water was added, and the solution was filtered to separate the acid from the solution. A single round of centrifugation was carried out, and dialysis was performed over a period of 2–3 days, as described earlier. The resulting suspension was stored in two phases.

The process for creating cellulose whiskers with oxalic acid was like that with citric acid, with the only difference being the use of a 70% concentration for 60–70 min at temperatures ranging from 100 °C to 110 °C. Preparation conditions and particle yields for each acid type are outlined in Table 1.

### 2.3. Cast Film Preparation

A solution comprised of 15 mL of distilled water and 3 g of PVA was placed in a beaker, along with a suspension of either CNC or CMF, each with a corresponding concentration of 1% and 5%. The mixture was heated to 80 °C with continuous stirring until the PVA was fully dissolved, which took approximately 40–60 min. The final solution was vacuum-filtered for 2 min to remove bubbles. Subsequently, the homogeneous mixture was cast onto polystyrene weighing dishes and left to dry in an oven at 40 °C overnight. The resulting films were then carefully removed and stored in a desiccator filled with sodium bromide at a relative humidity of 57% before undergoing characterization. The typical sample thickness was found to be within the range of 0.20 to 0.35 mm.

Due to the lower yield as indicated in Table 1 for CNC derived using the organic acids, cast films were prepared only with the CMF material obtained from the organic acid hydrolysis. Furthermore, for the sake of comparison, cast films were also prepared using CNC extracted from wood with sulfuric acid (obtained from the University of Maine), and these were compared with CNC obtained from hemp synthesized using sulfuric acid. The cast films containing cellulose nano crystals sourced from wood with concentrations of 1% and 5% were denoted as M-N-1 and M-N-5. The cast films produced in this project with cellulose nano crystals using sulfuric acid were labeled as S-N-1 and S-N-5. In addition, cast films containing 1% and 5% cellulose micro fibrils hydrolyzed with sulfuric acid, citric acid, and oxalic acid were labeled S-M-1, S-M-5, C-M-1, C-M-5, O-M-1, and O-M-5, respectively.

### 2.4. Characterizations

The yield of prepared cellulose whiskers was calculated using Equation (1) and is reported in Table 1.

(1) $$Yield\left(\%\right)=\frac{\left(m_{1}-m_{2}\right)\;v_{1}}{m_{3}{\;v}_{2}}\times 100$$

where m_2_ is the mass of the weighing dish (g), m_1_ is the total mass of the CNC/CMF powder and the weighting dish (g), m_3_ is the initial mass of hemp used (g), v_1_ is the total volume of CNC/CMF suspension synthesized, and v_2_ is the volume of the suspension analyzed [14].

#### 2.4.1. Morphological Characterization

A Scanning Electron Microscope (SEM) (FlexSEM 1000II, Hitachi High-Tech, Tokyo, Japan) was employed to investigate the morphology, shape, and structure of the cellulose whiskers and cast films. Additionally, for size measurements, the SEM images were evaluated using ImageJ software (Version 1.54h). In this technique, a drop of cellulose suspension in water (at a ratio of 1:10) was placed on aluminum foil and dried in an oven. Samples of the cast film were cut to dimensions of 8 × 8 mm and placed on carbon tape. High-voltage conditions of 15–20 kV were applied to capture the images. No significant differences in cast film morphology were observed.

#### 2.4.2. Fourier Transform Infrared Spectroscopy (FTIR)

The functional groups in the samples were analyzed by FTIR (NICOLET iS10 Smart iTR, Nicolet CZ, Prague, Czech Republic) to identify the molecular and intermolecular bonds present in the samples. The test was conducted for cellulose particle powders and small pieces of cast films in the mid-IR region, ranging from 4000 to 650 cm^−1^, at room temperature and a resolution of 4 cm^−1^, with an average of 64 scans.

#### 2.4.3. Thermal Analysis

Thermal degradation for both cellulose particle powders and cast films was investigated through thermogravimetric analysis (TGA) using a TGA Q50 instrument from TA Instruments (New Castle, DE, USA). The samples were heated to 600 °C at a rate of 10 °C/min in a N_2_ flow of 60 mL/min. Based on ASTM E2550–11 [33], the onset temperature was measured and reported as T_d_.

Differential scanning calorimetry (DSC) analyses using a DSC Q20 instrument, also from TA Instruments, were conducted to assess changes in the thermal properties of the cast films. The heat–cool–heat method was employed, cycling the temperature from 20 to 225 °C, which is lower than the thermal degradation temperature of the samples. The heating and cooling rates were set at 10 °C/min, and a N_2_ flow of 50 mL/min was maintained.

The degree of crystallinity was calculated as

(2) $$X_{c}=\frac{{∆H}_{m}}{w{∆H}_{0m}}$$

where ∆H_m_ represents the enthalpy of the sample film’s melting point, ∆H_0m_ is the enthalpy of melting for a 100% crystalline PVA sample (determined as 161.6 Jg^−1^ according to Roohani et al. [30]), and w signifies the weight fraction of PVA in the sample.

#### 2.4.4. Mechanical Properties of Cast Films

A universal tensile testing machine (INSTRON 5543A, Norwood, MA, USA) was utilized to measure the stress, strain at maximum load, and Young’s modulus of the cast films. The test was conducted at a crosshead speed of 17.5 mm/min and a gauge length of 50 mm, following ASTM D882 standards [34]. Ten film specimens for each sample, with a width of 5 mm, were tested under environmental conditions of 20 °C and 65% relative humidity.

## 3. Results and Discussion

### 3.1. CNC/CMF Characterization

#### 3.1.1. Morphological Characterization

The data presented in Table 1 are the highest yields achieved under optimal hydrolysis conditions. Citric acid hydrolysis yielded the highest total yield, with 95.5%, followed by sulfuric acid and oxalic acid, with 87.3% and 68.4%, respectively. Sulfuric acid hydrolysis produced the greatest quantity of CNC, at 41.9%, while oxalic acid exhibited a higher CMF yield compared to CMF produced with sulfuric acid. Furthermore, the percentage of CMF generated with citric acid surpassed that of the other acids, at 91.9%.

**Figure 1:**
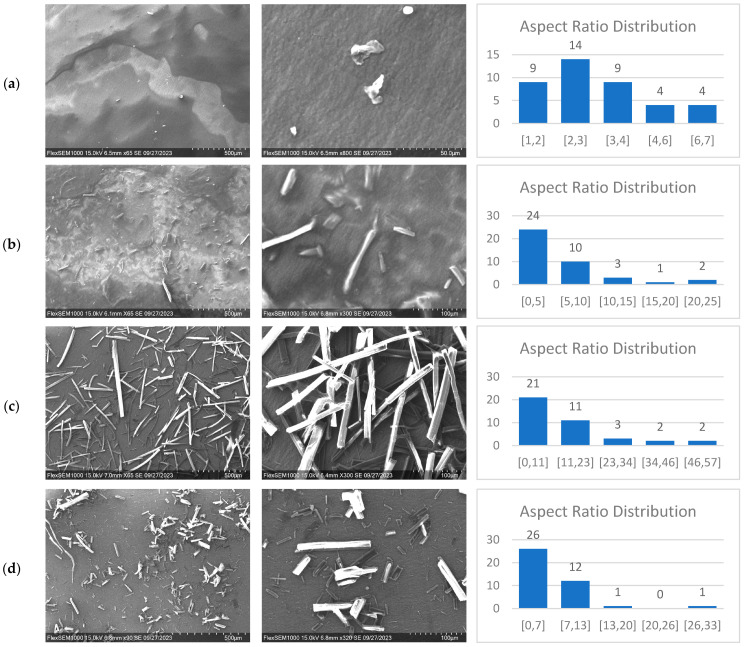
(**a**) SEM images and aspect ratio distribution of CNC using sulfuric acid, (**b**) SEM images and aspect ratio distribution of CMF using sulfuric acid, (**c**) SEM images and aspect ratio distribution of CMF using citric acid, (**d**) SEM images and aspect ratio distribution of CMF using oxalic acid.

In Figure 1 and Table 2, SEM data illustrate the various sizes and morphologies of cellulose particles resulting from the hydrolysis of hemp cellulose using different acids. Two levels of magnification were employed to provide a more comprehensive depiction of size, morphology, and distribution.

Post-hydrolysis, the cellulose nano crystals produced with sulfuric acid were thinner and shorter compared to the other particles. They exhibited shapes resembling needles, worms, and tiny spheres, as depicted in Figure 1a. Their sizes ranged from 20 to 300 nm in length and 15 to 100 nm in width. In contrast, the S-CMF particles, produced from the same process, exhibited microscale dimensions of 150 nm–20 μm in width and 300 nm–120 μm in length, with a longer needle-like shape and an aspect ratio of 2.5 (Figure 1b). Notably, the aspect ratio distribution of S-CNC and S-CMF was narrower than that of the other samples. These differences can be attributed to the preferential attack of hydrogen ions on the amorphous regions of cellulose, resulting in a rapid hydrolysis process that releases soluble sugars like cellobiose and polysaccharides [35].

CMF produced through hydrolysis with citric acid displayed a rod-shaped sheet structure, with lengths ranging from 1 to 400 μm, and the widest aspect ratio distribution, with a mean of 5.5. This could be due to fewer hydrogen ions targeting the amorphous regions, leading to a slower hydrolysis rate and the formation of larger particles (Figure 1c) [35].

The morphology of CMF produced with oxalic acid was a combination of shapes, including needle-like, worm-like, and small rod-like structures. These structures were longer than those generated with sulfuric acid but shorter than those created with citric acid, spanning a range from 900 nm to 140 μm, as seen in Figure 1d.

To summarize, the data from Table 1 and Table 2 indicate that citric acid yields the highest output, resulting in CMF with larger particle sizes and a wider aspect ratio distribution, characterized by rod-like shapes. Sulfuric acid generates the highest CNC yield, resulting in smaller sizes and a narrower aspect ratio distribution, with needle-like and worm-like morphologies. CMF produced with oxalic acid exhibits a variety of particle sizes and shapes, forming a mixture of needles and rods, with 62.5% CMF of the 68.4% total yield.

The variation in size and shape of cellulose particles across samples can be attributed to differences in the degree of hydrolysis. This is dependent on factors such as the type of acid used, whether it is a strong or weak acid, acid concentration, temperature, and process duration. Ultimately, the choice of whether to use organic or mineral acid for cellulose particle production clearly depends on the intended application.

#### 3.1.2. FTIR

**Figure 2:**
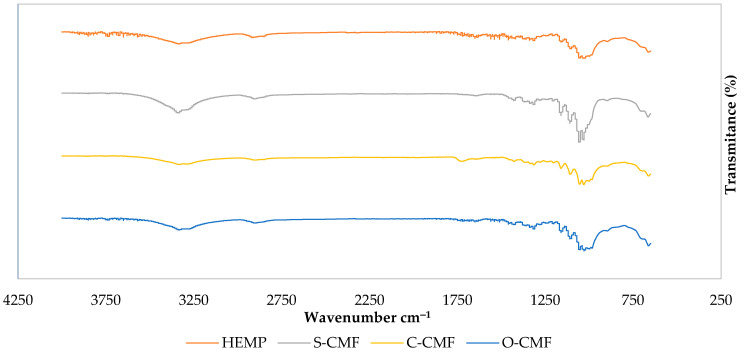
FTIR spectra of Hemp, S-CMF, C-CMF, and O-CMF.

The FTIR spectra of Hemp, S-CMF, C-CMF, and O-CMF are displayed in Figure 2 and Table 3 to compare the effect of the acid used in synthesis on cellulose particle chemistry.

In all samples, a broad peak around 3335 cm^−1^ indicates the O-H stretch in cellulose, and the subsequent peak near 2900 cm^−1^ represents the C-H groups, indicating the presence of the cellulose unit [36].

A distinctive peak at 1730 cm^−1^ is exclusively observed in C-CMF spectra, corresponding to the C=O stretch associated with the ester carboxylic or carbonyl group formed through the esterification process, involving the hydroxyl groups of cellulose and the carboxyl groups of citric acid. This surface modification has been reported in studies by Yuho et al. and Bondanisa et al. [32,37], providing evidence of successful surface modification. Conversely, the other samples exhibit small peaks around 1720 cm^−1^, representing the C=O stretching frequency related to the carbonyl group in hemicellulose and lignin [38]. Both can be associated with the use of oxalic acid.

An O-H group of adsorbed water around 1640 cm^−1^ is observed in the S-CMF and O-CMF spectrum, due to the interaction between adsorbed water and the hydrophilic surface group (-OH) of cellulose [39].

The absence of absorption bands at around the 1500–1600 cm^−1^ range in the C-CMF spectra (related to aromatic ring vibrations) indicate the removal of lignin in these microfibrils [40]. However, small extra peaks in this region can be observed for cellulose microfibrils made using sulfuric and oxalic acid.

The peak around 1430 cm^−1^ represents the O-CH_3_ bend for methoxyl-O-CH_3_ compounds in lignin, followed by a peak at approximately 1320 cm^−1^ associated with CH_2_ stretching from cellulose, signifying the exposure of the crystalline surface of cellulose resulting from the removal of non-cellulosic components in all samples [41,42], with a stronger intensity in S-CMF spectra compared to other spectra.

The absorption phenomenon in all spectra around 1205 cm^−1^ refers to the bending frequency of C-O phenol in lignin. Another possibility is attributed to the S=O stretching vibration from sulfate groups, demonstrating esterification of the hydroxyl groups, as seen in the S-CMF graph [43,44].

The absorption peaks around 1170 cm^−1^ and 1030 cm^−1^ are ascribed to C-O-C group vibrations of pyranose ring skeletal compounds in cellulose [45]. Similar peaks, but with higher intensity, are observed in the S-CMF spectra at around 1108, 1053, and 663 cm^−1^, corresponding to O-H group and C-O stretching, C-O deformation in the C-OH compound of lignin and hemicellulose, and C-C stretching, respectively, in all samples [43].

The spectral ranges of 3500–4000 cm^−1^ and 1400–1800 cm^−1^, associated with the stretching and bending vibration of hydroxyl groups, likely represent alkene, esters, aromatics, ketones, and alcohols in the degummed hemp [46]. However, these peaks disappear in all samples’ spectra, indicating the successful removal of hemicellulose and lignin following all acid hydrolysis treatments.

#### 3.1.3. Thermal Analysis

**Figure 3:**
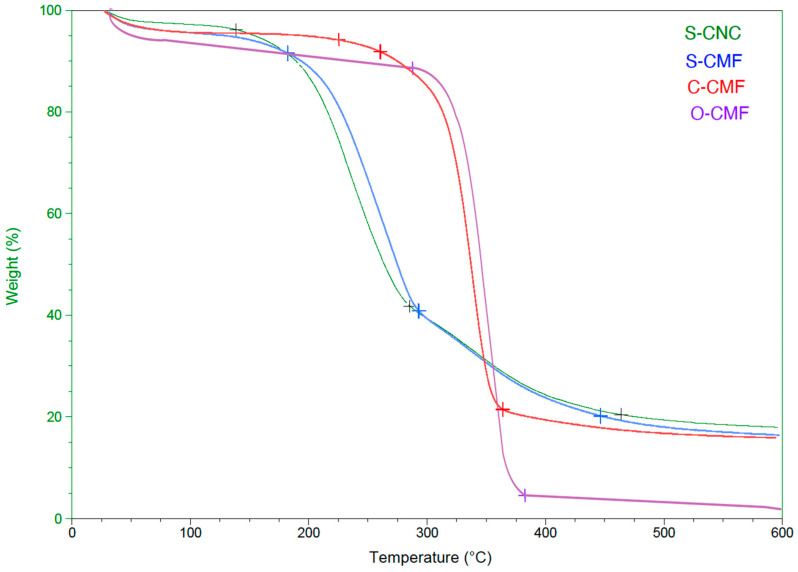
Thermogravimetric graph of S-CNC, S-CMF, C-CMF, and O-CMF.

Thermal degradation tests were conducted to assess the thermal stability of the materials. The obtained graphs indicated a negligible weight loss around 100 °C, signifying the removal of moisture along with the disruption of intermolecular hydrogen bonds in water [47,48]. It can be seen from Figure 3 and Table 4 that samples treated with sulfuric acid exhibited three main weight loss steps, in contrast to the two steps observed in samples treated with organic acids. The second weight loss step, characterized by the highest degradation peak within the temperature range of 150–290 °C for all samples, was attributed to cellulose depolymerization, dehydration, and glycosyl unit decomposition [49,50].

The third region, spanning from 290 °C to 460 °C for S-CNC and S-CMF curves, was associated with the breakdown of carbon-containing substances [51]. S-CNC showed the lowest decomposition temperature at 150 °C, losing less weight (around 80%) than the other three samples. S-CMF exhibited a thermal degradation point at 170 °C, with an 82% weight loss. This discrepancy may be attributed to reduced thermal stability linked to increased specific surface area and decreased dimensions [52,53].

Furthermore, the introduction of sulfated groups into the crystals during the acid hydrolysis process, as indicated by FTIR testing, could reduce the thermal stability of nanocrystals, as reported in earlier research [54]. These functional groups may weaken hydrogen bonds between adjacent cellulose chains and intramolecular hydrogen bonds, contributing to decreased thermal stability. CMF with oxalic acid exhibited a maximum degradation temperature of approximately 290 °C, showcasing excellent colloidal stability and the smallest interaction with water, thus achieving high thermal stability [12]. Conversely, sulfuric acid is known for its strong reaction with water. The curve for C-CMF shows considerable thermal stability, with less weight loss compared to O-CMF (86% vs. 95%).

Another factor contributing to the higher thermal stability of nanocellulose particles treated with organic acids compared to those treated with mineral acid is the presence of functional surface groups. For instance, citric acid hydrolysis of cellulose generates ester groups, releasing carboxylic groups on the nanocellulose surface, as evidenced by the FT-IR transmittance spectra at the 1730 cm^−1^ peak. This molecular bond is strong and highly resistant to breakage. Conversely, in the case of sulfuric acid, the hydroxyl groups on the CNC surface are randomly replaced by sulfate esters, leading to negatively charged S-CNC/CMF and relatively low thermal stability due to the presence of sulfate groups on the surface [55].

### 3.2. Cast Film Characterization

#### 3.2.1. FTIR

**Figure 4:**
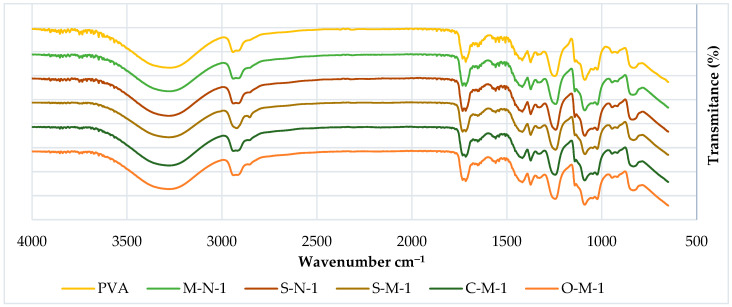
FTIR spectra of PVA cast films with 1% filler.

**Figure 5:**
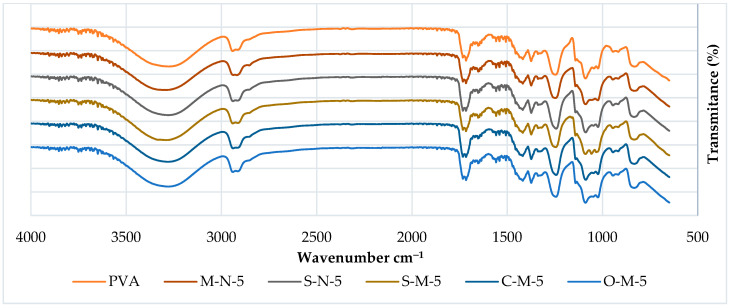
FTIR spectra of PVA cast films with 5% filler.

The chemical behavior of both neat PVA and PVA biocomposites was investigated through FTIR analysis, as illustrated in Figure 4 and Figure 5. The FTIR spectra of PVA reveal several prominent absorption bands. It is evident that all the spectra are similar, except for a few small changes. The peak at around 3274 cm^−1^ corresponds to the stretching vibration of (-OH), resulting from intramolecular hydrogen bonds within the PVA and intermolecular hydrogen bonding between hydroxyl groups of PVA and CNC. Notably, in cast films with reinforcement, the addition of CNC intensifies the peak at 3274 cm^−1^ [56].

Additional peaks at 2941 cm^−1^ (-CH stretching) arise from alkyl groups, and 1716 cm^−1^ (C=O stretching) is attributed to the C=O and C-O stretching from residual acetate groups in the PVA matrix. This peak shifts to a higher peak at 1732 cm^−1^ in cast films, indicating an increased number of C-O groups in the matrix, possibly due to the presence of hemicelluloses in the CNC. Another possibility could be the existence of a crosslinking structure between hydroxyl groups of PVA and CNC at around 1740 cm^−1^ [56] (though this has yet to be verified in our samples).

The absorption band at 1653 cm^−1^ corresponds to observed water molecules, while additional bands at 1418 cm^−1^ and 1374 cm^−1^ (C-H stretching) result from the bending vibration of C-H bonds, appearing in almost all spectra [57].

Furthermore, peaks were observed at 1373 cm^−1^, 1338 cm^−1^ (O-H stretching), indicative of hydroxyl crosslinking by hydrogen bonding of CNC/CMF, and 1247 cm^−1^ (-CO of carbonate). A double peak is observed in the region of 1089–1023 cm^−1^, where the peak at 1089 cm^−1^ (-O-C=O carbonate) corresponds to CO stretching vibration, and the peak at 1023 cm^−1^ could be attributed to the CH bending vibration of CH_2_ groups. The second peak at 1023 cm^−1^ becomes sharper with the addition of CNC, reducing the intensity of the first peak at 1086 cm^−1^, indicating a potential interaction between PVA and the cellulose particles [58,59].

Another double peak is observed around 917 cm^−1^ (C-H stretching bonding), as reported by Alemdar and Sain, 2008a, which undergoes a shape change and shifts to a higher peak at around 945 cm^−1^ in the composite compared to neat PVA. Additionally, an absorption band at 833 cm^−1^ (C-C methyl group) is gradually reduced with the increase of CNC, supporting the possible interaction between CNC and PVA [60,61].

It is noteworthy that in S-M-5, three peaks are observed instead of a double peak around 1058 cm^−1^, and the spectra of S-M-1 present a single peak instead of two, with a smaller peak at 2822 cm^−1^.

#### 3.2.2. Thermal Analysis

##### TGA

Thermogravimetric analysis was conducted to assess the thermal stability of PVA and biocomposite cast films, as reported in Table 5. No discernible trend was identified among all samples; however, enhanced thermal stability was observed in samples incorporating CMF and 5 percent of CNC.

A consistent pattern of two main weight losses was observed across all samples. A minor reduction with small weight loss in the 100–150 °C range was noted, attributed to the evaporation of moisture or low molecular weight compounds [62]. The peak temperature corresponding to the primary decomposition step for neat PVA and PVA/CNC/CMF biocomposites fell within the range of 257–271 °C. Notably, O-M-1 exhibited the highest thermal stability, followed closely by S-M-1 and C-M-1, with temperatures of 271 °C, 270 °C, and 269 °C, respectively. With the addition of cellulose particles (5%), the rate of weight loss increased. S-N-1 displayed the minimum weight loss at 54 percent, approximately 40% less than the others. Consequently, the incorporation of CMF and CNC with 5% in the PVA matrix resulted in only a slight enhancement in the thermal stability of the matrix reinforcement.

##### DSC

Differential scanning calorimetry was employed to assess the glass transition temperature (T_g_), melting temperature (T_m_), and crystallization temperature (T_c_) of pristine PVA and biocomposite films reinforced with cellulose particles, as detailed in Table 5. To eliminate moisture and ensure consistent thermal history, all samples underwent a 30 min treatment at 130 °C.

Common thermal behavior patterns were observed among all samples, featuring two endothermic peaks in the second heating cycle and one exothermic peak during cooling. However, the thermal response exhibited no consistent pattern following the incorporation of CNC/CMF in the PVA cast film. A noteworthy increase in specific heat around 66 °C, corresponding to the glass–rubber transition (T_g_) of PVA, was observed. While the glass transition temperature of PVA experienced a slight decrease with the addition of reinforcement, this effect was more pronounced at higher CNC and CMF contents. However, only the O-M-5 sample demonstrated a higher T_g_ compared to PVA, increasing from 66 to 68 °C. The initial reduction in T_g_, attributed to the restriction of PVA chain mobility due to the adsorptive forces of the reinforcement, aligns with previous findings [57,63].

The melting temperature of PVA showed an increase of approximately 10 °C for all samples with both 1% and 5% reinforcement. C-M-5 displayed a slight increase to 179 °C compared to neat PVA at 175 °C, whereas O-M-5 exhibited the maximum T_m_, at 188 °C.

Table 5 illustrates a slight enhancement in the crystallinity of cast films with the addition of 1% and 5% percent of CNC and CMF. M-N-1 and S-M-5 demonstrated a higher increase in crystallinity, reaching 18% compared to pure PVA (11%). This rise in crystallinity is attributed to the nucleating effect of nano-sized fibers, supporting previous studies [29].

A parallel trend was observed in the crystallization temperature during the cooling scan with the increased concentration of CNC/CMF. This elevation in T_c_, associated with cellulose particle addition, is attributed to variations in crystal size and morphology in cast films. The PVA membrane with more uniform crystals experiences dominant homogeneous phase nucleation during cooling [63].

### 3.3. Mechanical Properties of Cast Films

**Figure 6:**
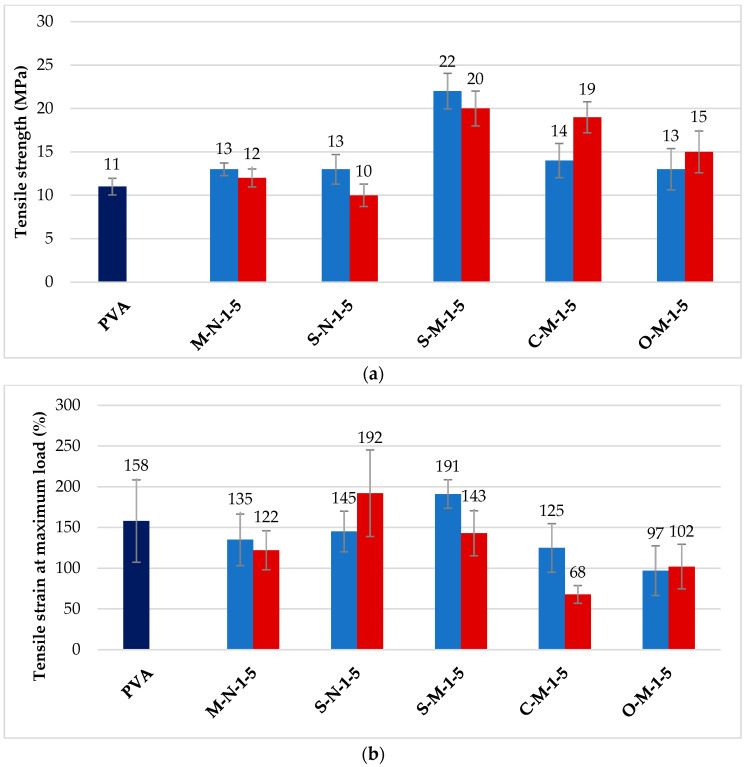

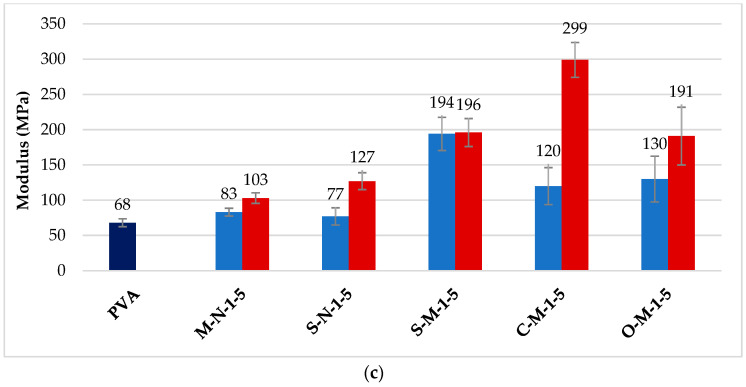
(**a**) Tensile strength, (**b**) tensile strain at maximum load, (**c**) Young’s modulus of unreinforced PVA and 1% and 5% reinforced biocomposites.

Figure 6a displays the tensile strength results of PVA and the reinforced films with nano and micro cellulose at 1% and 5% reinforcement. The light blue is for films with 1% reinforcement, and the red bar is for films with 5% reinforcement. It is observed that the tensile strength for all reinforced films increased compared to unreinforced PVA (dark blue), except for the sample with 5% CNC derived from hemp using sulfuric acid. For the other samples, there is an overall enhancement in the film’s tensile strength, consistent with previous studies [64,65]. It has been mentioned that the strong affinity between the PVA matrix and CNC/CMF, both carrying hydroxyl-rich surfaces, makes for relatively strong interfacial interactions [30]. These interactions, characterized by hydrogen bonding, significantly strengthen the interface, impacting the mechanical properties of the composite material [1,29]. This enhancement is more pronounced in CMF-reinforced samples as compared to CNC-reinforced samples, with strengths approximately 2, 1.7, and 1.3 times larger for CMF-reinforced samples prepared with sulfuric acid, citric acid, and oxalic acid, respectively. This could be attributed to the larger particles present in these samples, as shown in Figure 1, with higher aspect ratios. Furthermore, Lee et al. (2009) [66], described that using 1 wt% of cellulose nanocrystals led to an increase in tensile strength, but when the CNC loading was increased to 5 wt% in a PVA matrix, the tensile strength slightly reduced, from 13 to 12 MPa and from 13 to 10 MPa for CNC with wood and CNC with hemp, respectively. This behavior is not observed in the case of CMF samples due to differences in particle characteristics, such as size, aspect ratio, crystallinity, morphology, and distribution, which can impact the mechanical properties [67].

The tensile strain results are shown in Figure 6b, where the dark blue bar is the PVA control, the light blue bars are composites with 1% reinforcement, and the red bars are composites with 5% reinforcement. As observed in the graph, the tensile strain of most samples, except for two, decreased when compared with the unreinforced PVA film. The minimum strain, 68%, is observed in the case of C-M-5. This significant decrease is due to the carboxylate bonds at the fiber interface, which make the interface stronger but more brittle. On the other hand, the maximum strain, 192%, is observed in the case of S-N-5 and S-M-1. These values indicate more plastic behavior, with extensive plastic deformation and slippage at the fiber–matrix interface. Therefore, depending on the specific application, the appropriate sample can be selected to achieve the desired mechanical response [68,69].

In Figure 6c, the impact of cellulose nano/micro particles on the Young’s modulus is demonstrated. The color legend remains the same for this graph. The results indicate that the inclusion of cellulose nanocrystals and cellulose microfibrils as reinforcement leads to an increase in the modulus. The modulus increases with the addition of CNC/CMF loading, and films with CMF exhibit a higher modulus compared to CNC films, ranging from 120–299 MPa to 77–127 MPa, respectively. When the CMF concentration is 5% with citric acid, the modulus obtains its maximum value, which is 4.4 times greater than that of the unreinforced PVA film. It is important to note that these increases in modulus are significantly higher than one would expect with 1% and 5% reinforcing particles using models such as the rule-of-mixtures or the Halpin–Tsai equations. This increase can be attributed to the stiffening of the polymer matrix due to chain mobility constraints introduced by the large fiber–matrix interface introduced by the addition of micro-fibril reinforcements [64,70].

These results demonstrate a significant improvement in mechanical properties through the addition of CNC/CMF in these biocomposites. It is worth noting that one of the key considerations for such composites with natural reinforcement is uniform particle distribution, which can have an impact on mechanical properties.

## 4. Conclusions

This study focused on the production of cellulose whiskers from hemp through the application of both organic and mineral acids. The first step involved characterizing cellulose particles derived with different acids to compare their properties. Morphological and thermal analyses, supported by chemical analysis, revealed that cellulose particles derived from hydrolysis with citric acid had the highest total yield at 95.9%, followed by 62.5% CMF using oxalic acid. In contrast, using sulfuric acid produced the highest yield of nanocrystals at 41.9%. Particles synthesized using citric acid exhibited the highest aspect ratio, with larger rod-like particles, while particles synthesized using sulfuric acid produced cellulose particles in the nano range with needle and worm-like shapes. In terms of thermal stability, particles synthesized using organic acids were significantly more stable compared with particles synthesized using sulfuric acid, which exhibited poor thermal resistance due to weaker bonds with water and esterification of the sulfate surface groups present in sulfuric acid.

Characterizing cellulose fillers in biocomposite films involved several tests. The morphology of cast films exhibited consistent surface modification. FTIR results confirmed interfacial forces between the filler and the polymer. In terms of thermal properties, the addition of the reinforcements to PVA increased the melting and crystallization temperatures and increased the degree of crystallinity compared to unreinforced PVA. The thermal stability slightly increased in nearly all reinforced samples.

Tensile tests yielded significant results. Tensile strength and Young’s modulus increased in almost all reinforced samples, with CMF-containing samples showing more significant increases. Reinforcing with particles synthesized using citric acid exhibited the highest modulus, with an increase of more than four times the value for unreinforced PVA. This composite also demonstrated the lowest strain to failure due to the presence of carboxylic groups at the fiber–matrix interface, making for a stronger but more brittle interface. The yielded composite could be optimized for applications such as food packaging, dissolving medical applications, or other disposable plastic items. Further optimization of the manufacturing of hemp CNCs should be performed before downstream applications are considered. Without further modification of the particle and the PVA matrix, only applications where water absorption is tolerable can be considered.

The dispersion of these fillers into the composite and its impact on the results will be further investigated using Dynamic Light Scattering (DLS) in future studies. Evaluation of the crystallinity of the PVA composites is currently underway using X-Ray Diffraction (XRD). Additional tests to measure the zeta potential will be conducted to complete the characterization of these cellulose particles synthesized from organic and mineral acids. Measuring zeta potential will help to analyze the fiber–matrix interface and will be complemented with surface energy measurement using experiments such as the Wilhelmy single-fiber wetting technique.

## Figures and Tables

**Table 1:** Synthesis of CNC/CMF: reaction temperatures, T (°C); processing times, t (min); acid concentrations, *w*/*w* (%); and yields (%).

Samples	T (°C)	t (min)	*w*/*w* (%)	CNC Yield (%)	CMF Yield (%)	Total Yield (%)
Sulfuric Acid Hydrolysis	65–70	60	64	41.9	45.5	87.3
Citric Acid Hydrolysis	95–105	240	80	3.7	91.9	95.5
Oxalic Acid Hydrolysis	100–110	60–70	70	5.9	62.5	68.4

**Table 2:** The length, width, and aspect ratio (L/W) of CNC/CMF synthesized using different acids.

Samples	Length		Width		(L/W)
	Range	Mean	Range	Mean	Mode
S-CNC	20–300 nm	100 nm	15–100 nm	60 nm	2.5
S-CMF	300 nm–120 μm	20 μm	150 nm–20 μm	5 μm	2.5
C-CMF	1–400 μm	100 μm	300 nm–22 μm	7 μm	5.5
O-CMF	900 nm–140 μm	23 μm	200 nm–20 μm	5 μm	3.5

**Table 3:** Summary of significant FTIR peaks in Figure 2 (described above).

PEAK	HEMP	S-CMF	C-CMF	O-CMF	BOND SOURCE
3335	X	X	X	X	O-H (cellulose)
2900	X	X	X	X	C-H (cellulose)
1730			X		C=O (ester)
1720	X	X		X	C=O (carbonyl)
1640		X		X	O-H (water)
1500–1600	X	X		X	aromatic ring (lignin)

**Table 4:** Degradation temperature (T_d_), moisture content (MC), and mass change of CNC with sulfuric acid (S-CNC), CMF with sulfuric acid (S-CMF), CMF with citric acid (C-CMF), and CMF with oxalic acid (O-CMF).

Sample	T_d_ (°C)	MC (%)	Mass Change (%)
S-CNC	150	4	80
S-CMF	170	6	82
C-CMF	230	5	86
O-CMF	290	9	95

**Table 5:** Glass transition temperature (T_g_), melting temperature (T_m_), heat of fusion (∆H_m_), crystallinity degree (X_c_), crystallization temperature (T_c_), degradation temperature (T_d_), moisture content (MC), and Total Mass Change (TMC) of PVA and the reinforced PVA films.

Samples	T_g_ (°C)	T_m_ (°C)	∆H_m_ (J/g)	X_c_ (%)	T_c_ (°C)	T_d_ (°C)	MC (%)	TMC (%)
PVA	66	175	18	11	127	264	6	92
M-N-1	60	188	28	18	153	257	7	95
S-N-1	65	185	22	14	147	261	4	59
S-M-1	60	187	22	14	154	270	10	90
C-M-1	60	188	27	17	155	269	5	89
O-M-1	62	186	21	13	149	271	6	99
M-N-5	48	185	26	17	153	264	13	94
S-N-5	59	187	23	15	163	266	17	96
S-M-5	55	186	27	18	158	269	12	96
C-M-5	61	179	20	13	142	269	14	97
O-M-5	68	188	19	12	150	258	11	99

## Data Availability

All data are presented in the manuscript.
